# Carcinoma with Triphasic Differentiation Arising from Inverted Papilloma in Sinonasal Sinus: A Rare Case with Molecular Characterization

**DOI:** 10.3390/diagnostics11101827

**Published:** 2021-10-03

**Authors:** So-Woon Kim, Kiyong Na

**Affiliations:** Department of Pathology, Kyung Hee University Hospital, College of Medicine, Kyung Hee University, Seoul 02447, Korea; sowoonkim86@gmail.com

**Keywords:** small cell neuroendocrine carcinoma, sarcomatoid carcinoma, squamous cell carcinoma, sinonasal sinus, composite tumor

## Abstract

Small cell neuroendocrine carcinoma (SNEC) is a rare subset of tumors in the sinonasal sinus. Combined tumors are exceedingly rare. Here, we describe a 65-year-old male with a mixed tumor of SNEC and sarcomatoid carcinoma arising in an inverted papilloma, containing squamous cell carcinoma in situ (SqCCis) in the sinonasal sinus. We evaluated the molecular characteristics of the two separate carcinoma components using next-generation sequencing. The patient presented with a nasal obstruction. Computed tomography showed a mass infiltrating the right ethmoid and maxillary sinuses. An excisional biopsy was performed. The tumor was found to have three morphologically distinct components. The first was SqCCis arising in an inverted papilloma, which was positive for cytokeratin and P40. The second consisted of nests of densely packed small round cells representing SNEC-positive neuroendocrine markers. The third was a solid sheet of anaplastic spindle cell proliferation, which was negative for the above markers. Oncogenic mutations such as *FBXW7, TP53*, and *EGFR* were detected in both SNEC and sarcomatoid carcinoma, and *MYCL* amplification was observed only in the SNEC component. This case highlights an extremely rare presentation of combined SNEC and sarcomatoid carcinoma arising from an inverted papilloma in the sinonasal sinus.

Tumors of the sinonasal tract are rare, representing approximately 3% of head and neck malignancies and approximately 0.3% of all malignant tumors [1]. The most frequent histologic subtype is squamous cell carcinoma (SqCC), followed by adenocarcinoma, melanoma, and olfactory neuroblastoma [1,2]. Small cell neuroendocrine carcinoma (SNEC) is a rare tumor in the head and neck region that occurs most frequently in the larynx, while sinonasal sinuses are exceedingly rare primary sites [3].

Only a small case series and case reports have been published regarding primary sinonasal tract SNEC [3]. In addition, only a few examples of combined tumor have been reported in the sinonasal tract. These consisted mainly of a combination of adenocarcinoma and neuroendocrine carcinoma, while cases of combined small cell and SqCC appear to be very rare [1,4]. Furthermore, the collision of three components (neuroendocrine cells, sarcomatous cells, and squamous cells) in a solid tumor is extremely rare, and no reports of combined tumors with these triphasic features have been reported in the literature [2].

Here, we describe the clinicopathological, immunohistochemical and molecular features of primary combined SNEC and sarcomatoid carcinoma arising from an inverted papilloma containing squamous cell carcinoma in situ (SqCCis) in the sinonasal sinus.

Next, we performed next-generation sequencing on each SNEC and the sarcomatoid carcinoma component to analyze the molecular heterogeneity of the tumor. As a result, oncogenic mutations, such as *FBXW7* (c.1972C>T, p. R658*), *TP53* (c.740A>T, p. N247I), and *EGFR* (c.2319_2320insAACCCCCAC, p. D770_N771insNPH) were detected in both the SNEC and sarcomatoid carcinoma components. Of note, *MYCL* amplification was observed only in the SNEC component, a frequent oncogenic event in SNEC, supporting our diagnosis. [5]. *FBXW7* is well known to be highly frequently mutated in SqCC, and a loss of *TP53* and mutation of *FBXW7* in SqCC are known to lead to resistance to standard chemotherapy [6,7]. Therefore, it seems that this heterogeneous tumor arises from a single SqCC component and generates SNEC through further molecular alterations. The final stage was T4bN0M1.

After the biopsy, the patient underwent radiation therapy of 70.2 Gy. However, six months after surgical resection and radiation therapy, follow-up brain CT showed new round masses abutting the superior falx cerebri, left parietal lobe, and both frontal lobes (Figure 1D). The patient died later that month.

This case highlights an extremely rare presentation of primary combined SNEC and sarcomatoid carcinoma arising from an inverted papilloma in the sinonasal sinus. To the best of our knowledge, there have been no cases of combined SNEC and sarcomatoid carcinoma diagnosed in the sinonasal sinus reported in the literature. Since it can affect the treatment plan of chemotherapy or radiation therapy depending on the components constituting the tumor, the need for accurate pathological diagnosis and technology is required. Our findings can help pathologists and clinicians make accurate histological diagnoses of combined SNEC and sarcomatoid carcinoma and plan an adequate treatment strategy for this rare tumor.

The stains used were hematoxylin and eosin stain (Figure 2 and Figure 3) and the polymer method (Figure 3). The original magnifications were 40× in Figure 2, 400× in the inset of Figure 2, and 200× in Figure 3.

## Figures and Tables

**Figure 1:**
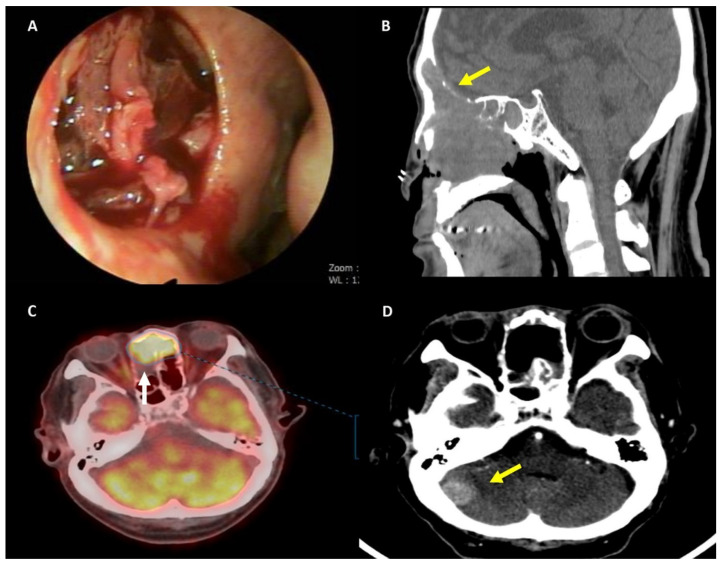
A 65-year-old male was referred to the department of otorhinolaryngology with epistaxis and right nasal obstruction for two months. His medical history revealed diabetes mellitus, hypertension, and pulmonary tuberculosis. (**A**) Physical examination demonstrated an easy-bleeding tumor in the right nasal cavity and bloody crust with an ulcerative lesion at the anterior septum; (**B**) The initial computed tomography (CT) showed a 4.5 cm × 3.4 cm × 4.8 cm soft tissue lesion infiltrating the right ethmoid and maxillary sinuses with destruction at the frontal sinus posterior wall and cribriform plate. Magnetic resonance imaging (MRI) showed a residual mass with destruction in the posterior wall of the frontal sinus, cribriform plate, and dura invasion with suspicious brain parenchyma (right frontal lobe) invasion; (**C**,**D**) Fluorodeoxyglucose positron emission tomography (FDG-PET) showed a residual hypermetabolic mass involving the superior portion of the nasal cavity and the anterior portion of the ethmoid sinus, with a maximum standardized uptake value (SUVmax) of 8.5. Clinical differential diagnoses included inverted papilloma with malignant transformation, lymphoma, and olfactory neuroblastoma. To confirm the diagnosis, an excisional biopsy was conducted.

**Figure 2:**
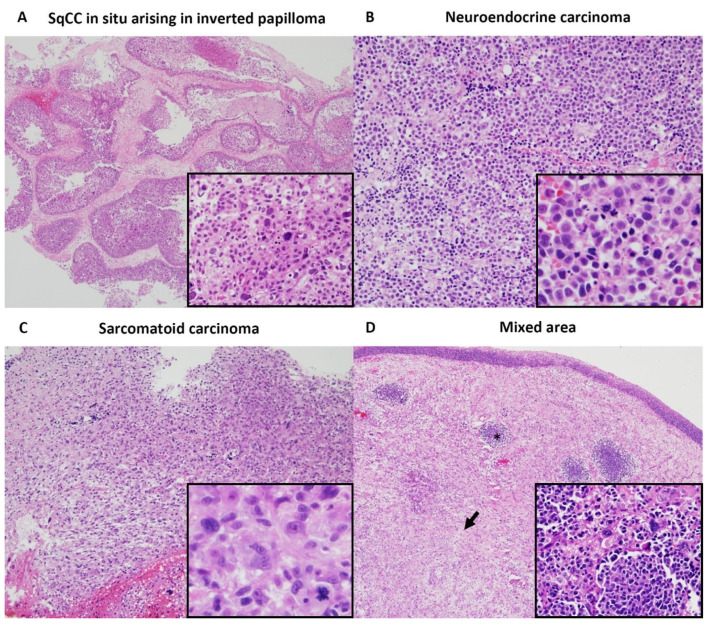
Endoscopic excisional biopsy of the right nasal cavity mass was performed. The tumor was found to consist of three morphologically distinct components in the resected specimen. These components were partly clearly separated and partly strictly mixed. (**A**) The first component was SqCCis arising in inverted papilloma, which represented approximately 20% of the lesion. (**B**) The second component comprised of nests of closely packed, small round to atypical oval cells, with hyperchromatic nuclei, coarse chromatin, and scant pale eosinophilic cytoplasm. The tumor had karyorrhectic debris and mitotic activity. (**C**) This represented about 40% of the lesion. The third component consisted of a solid sheet of anaplastic spindle cells proliferation with prominent mitosis and multinucleated giant cells, which represented the remaining 40% of the lesion. (**D**) Of note, the focal area showed a tumor with admixed small round cell nests and anaplastic spindle cell elements overlying SqCCis. Asterisk, small round cell nest; arrow, spindle cell proliferation; right upper, squamous cell carcinoma in situ. The original magnifications were 40× and 400× in the inset

**Figure 3:**
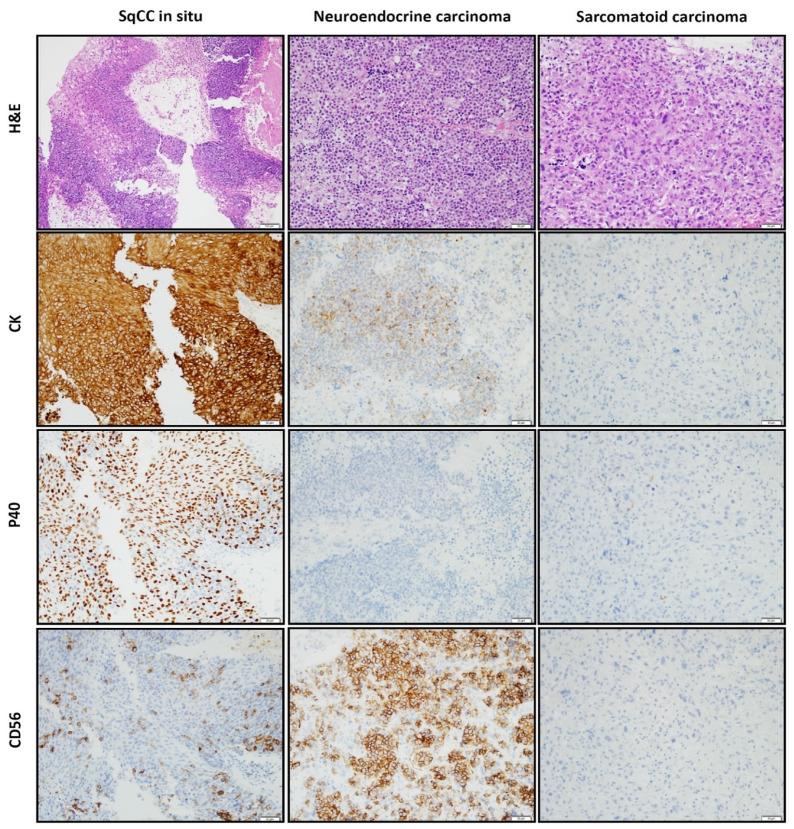
To characterize each component, an immunohistochemical examination was performed. The results of the immunohistochemical studies are illustrated in Figure 3. (**Left column**) The three components clearly showed differences in their immunoprofiles. SqCCis was diffusely cytoplasmic positive for pan-cytokeratin (CK) and nuclear positive for P40, but negative for the neuroendocrine markers CD56, synaptophysin, and chromogranin. Conversely, the nests of atypical small cells were positive for CK with a perinuclear dot pattern and diffusely cytoplasmic positive for the neuroendocrine markers CD56, synaptophysin, and chromogranin. (**Middle column**) However, the nests were negative for P40. (**Right column**) The anaplastic spindle cell lesion was negative for all markers. Based on the histologic features and the results of the immunohistochemical studies, a diagnosis of combined SNEC and sarcomatoid carcinoma arising in an inverted papilloma was rendered. It is important to distinguish whether a spindle cell lesion is another sarcoma or a spindle cell variant of SqCC (sarcomatoid carcinoma). A diagnosis of sarcomatoid carcinoma can be considered if the existing conventional squamous cell carcinoma is mixed with the spindle cell lesion or if the SqCCis is present together [8,9]. In our case, because the spindle cell component coexisted with SqCCis, we diagnosed this component as sarcomatoid carcinoma rather than another sarcoma. The original magnifications were 200×.

## Data Availability

The data presented in this study are available upon request from the corresponding author. The data are not publicly available because of privacy and ethical restrictions.
